# Editorial

**Published:** 1872-02-01

**Authors:** 


### MEDICAL EXAMINER.

*A Semi-Monthly Journal of Medical Sciences.*

EDITED BY

N. S. DAVIS, M. D., AND F. H. DAVIS, M. D.

Chicago, February 1st, 1872.

### EDITORIAL.

THE GAZETTE HEBDOMADAIRE, for Jan. 5th, 1872, contains quite an extended and very favorable editorial comment upon the "Medical Declaration of the London Physicians Respecting Alcohol."

A full report is also given of a highly interesting *memoire*, presented to the Academy of Medicine on December 25, on the subject of "Temperance Societies, and the possibility of their formation in France," by M. le Doctor A. Faville, jr. He first gives a complete review or history of the temperance movement, commencing with the formation of the American Temperance Society, at Boston, in 1827, and following its rapid extension, from year to year, throughout this country and Great Britain. Mention is also made of Dr. Rush, to whom the honor is accorded of having made the initiatory efforts in this great cause, as early as 1804, in the publication, at Philadelphia, of his "Inquiries into the effects of ardent spirits upon the body and mind."

After explaining the plan of organization and mode of action of our temperance societies, he speaks as follows, in regard to the results of their working: "The results of the work of these organizations it is most difficult to determine with accuracy. The influence extended by them being mostly of a moral character escapes all statistics, and can be neither weighed nor measured. Without doubt, the high and generous hopes of their first founders have not been realized. More than a generation has passed away since the initiation of the temperance movement, and there are drunkards still. Intemperance has been a scourge in all times and in all countries, and the war against it must be continuous and permanent. The temperance societies so far from being discouraged have redoubled their efforts to extend their influence and to increase the number of their adherents. These efforts, both in England and America, have not been without their results. We believe it is impossible to state exactly the number of *teetotalers* there are to-day in those countries, but they undoubtedly number many thousand, and probably millions. The working classes tend more and more to divide into two classes—those who drink to excess and those who do not drink at all. The latter, who are called *temperance men*, are easily distinguished by their good health, their neat appearance and decorous manners, and the confidence and esteem that they always elicit from those who employ them."

As regards the introduction of the temperance movement into France the writer does not think that the *total abstinence* principle would ever meet with favor there. First, because the moderate use of light wines, beer or cider with the ordinary meals is a universal national custom; secondly, because the cultivation of the grape forms so large and important a branch of the agricultural industry of the country; and, thirdly, he does not consider the moderate use of these weaker stimulants, taken with the ordinary meals, as in any way injurious, but, on the contrary in many cases, positively beneficial. This is the view advocated by everybody—physicians included—not only in France but in Germany and all the continental countries. At the same time, the habit of the excessive use of alcoholic stimulants is looked upon as being quite as much of a disgrace in those countries as in our own. The evils of intemperance are universally recognized in all countries.

With a view of suppressing or controlling this great vice M. Faville recommends the establishment, throughout France, of societies or *legues* similar in their organization to our temperance societies, but founded on the principal of total abstinence from all the stronger spirituous liquors, and the avoidance of the frequenting of *cafes* and public drinking saloons, but allowing of the use, in moderation, of the ordinary wines and beers at home, or with the regular meals.

These principles are such as would meet with favor from all the more intelligent, sober and industrious people not only in France but in all other European countries.

MEDICAL OPINION IN ENGLAND REGARDING USE OF ALCOHOL.—It is with much satisfaction that we have noticed, for some time past, evidences of a better appreciation of the relation of alcoholic drinks to medical practice, both in this country and in Europe.

In a recent number of *The Doctor*, of London, we find the following very important declaration of opinion signed officially and in detail by several hundred physicians, embracing a large majority of the most eminent members of the profession both in London and the provincial towns. The document is as follows:

"MEDICAL DECLARATION RESPECTING ALCOHOL.—As it is believed that the inconsiderate prescription of large quantities of alcoholic liquids by medical men for their patients has given rise, in many instances, to the formation of intemperate habits, the undersigned, while unable to abandon the use of alcohol in the treatment of certain diseases, are yet of opinion that no medical practitioner should prescribe it without a sense of grave responsibility. They believe that alcohol, in whatever form, should be prescribed with as much care as any powerful drug, and that the directions for its use should be so framed as not to be interpreted as a sanction for excess, or necessarily for the continuance of its use when the occasion is past.

"They are also of opinion that many people immensely exaggerate the value of alcohol as an article of diet, and since no class of men see so much of its ill effects, and possess such power to restrain its abuse, as members of their profession, they hold that every practitioner is bound to exert his utmost influence to inculcate habits of great moderation in the use of alcoholic liquids.

"Being also firmly convinced that the great amount of drinking of alcoholic liquors among the working classes of this country is one of the greatest evils of the day, destroying—more than anything else—the health, happiness and welfare of those classes, and neutralising, to a large extent, the great industrial prosperity which Providence has placed within the reach of this nation, the undersigned would gladly support any wise legislation which would tend to restrict, within proper limits, the use of alcoholic beverages, and gradually introduce habits of temperance."

Nearly thirty years since, we commenced an earnest and thorough investigation into the effects of alcohol on the human system, and the rational indications for its use in the treatment of diseases. We were soon led, both by experiments and clinical observation, to the conclusion that the professional and popular ideas in regard to the tonic, nutritive and supporting effects of alcoholic drinks were entirely erroneous; and that their necessary or beneficial use in medical practice is very limited indeed. We were, also, long since convinced that the medical profession were largely responsible for all the evils flowing from the use of alcoholic drinks among civilized nations. That the habit of recommending patients to take some one of the alcoholic drinks without careful limits as to time and quantity has been the direct means of starting an appetite that has led many to the drunkard's goal, is doubtless true. But the custom, so generally indulged in by the members of the profession everywhere, of speaking of these drinks as restorative, supporting and stimulant, in their daily intercourse with all classes of society, has impressed these erroneous ideas indelibly upon the public mind; and no amount of either moral or legal suasion will stop the habits of drinking and consequent drunkenness, until our profession reverses its teaching and thoroughly revises its practice in regard to these agents. As a step in the right direction, we hail the above declaration of our professional brethren in England with great pleasure. We are glad to learn that the same subject is receiving favorable attention also on the continent of Europe, as the reader will see by another paragraph in the present number of the EXAMINER.

A MEDICAL EXHIBITION.—We call the special attention of our readers to the following circular, which we have received, relating to an Annual Exhibition in connection with the next meeting of the American Medical Association. The circular explains itself, and is as follows:

"The undersigned, Chairman and Secretary of the Committee of Arrangements of the

American Medical Association, have been authorized to invite attention to the project of an exhibition of objects interesting to the medical profession, to be held in Philadelphia during the next session of the Association. This exhibition has been suggested as a desirable amplification of what has been customary on these occasions; and is expected to resemble, more or less, the displays of this kind which are prominent features of the annual meetings of the British Medical Association.

"In accordance with this determination to have an exhibition on the 7th, 8th, 9th and 10th of May, 1872, during the next session of the Association, the Committee of Arrangements would respectfully and earnestly appeal for contributions of objects to be exhibited—and for other available co-operation—to members of the medical profession, pharmacists, and manufacturers of chemicals, to opticians, instrument makers, publishers and booksellers, and to all others who are concerned in manufacturing or dealing in anything related to the study and practice of medicine and surgery and the associate sciences.

"They will gratefully receive choice specimens and examples (likely to prove interesting through novelty, rarity, importance or superior character) of drugs, medicines, and other remedial appliances—including special chemicals and pharmaceutical compounds and materials—as well as the apparatus employed in pharmaceutical and chemical processes; also of optical and other instruments of observation and precision; of surgical instruments and implements; of preparations and objects in natural history, including human and comparative anatomy, morbid or healthy; of models, drawings, paintings, prints, and of printed works—of recent date or standard character—on medicine, surgery, and the associate sciences.

"The views of the committee, in regard to the materials and the general nature and purpose of the exhibition, may be understood from the following paragraphs of the original announcement:

"'The aim of the committee will be, in this attempt, to provide for the practical and scientific entertainment of the members of the Association. Their design is to form a collection of instruments, apparatus, specimens, preparations, models, drawings, plates, books, and all other proper objects that may be obtained or presented in good time for the purpose; and so to arrange it for exhibition as to bring it under the convenient observation of every delegate and professional visitor. They hope, in this manner, without any sacrifice of the usual regard for their guests, to give to the arrangements as much of a professional character as may be in their power. Their desire is to aid in advancing the practical interests of the Association, by affording, through an always useful channel, some more direct means, as well as signs, of technical and scientific progress, as an attractive addition to the ordinary routine of written and verbal communications and discussions.'

"'They cannot promise much success in a first experiment—undertaken at unavoidably short notice—beyond that of the pioneer in preparing the way for something better in the future, in the light of experience, and with more time and opportunity for concerted action.

"'No mere display of local wealth and variety of means and appliances, or of individual superiority, will be encouraged beyond what is entirely incidental to the general purpose; nor will there be any attempt on the part of the committee to present reprentative or historical collections, although such collections may be cordially welcomed. Competition and completeness, therefore, are not to be expected. No special reports or comparative statements need be looked for; nor will the committee be responsible for the merits or demerits of the several objects exhibited, although obliged to exercise control as to admission and location. Novelty, rarity, and practical character will necessarily have weight in determining precedence; but not at the expense of whatever may be deemed especially characteristic or interesting, whether old, rare, or new. In a word, under their limitation of time, space and means, they cannot undertake a general exposition or an industrial fair. Further and more specific details will be published as soon as practicable.

"'The committee confidently hope for encouragement and assistance, in an early practical response from their professional brethren, and from others who may have objects of interest to offer. They are bound to remind all concerned, however, that the contemplated collection must of necessity be, as much as practicable, select and characteristic rather than varied and extensive. It ought to be comprehensive, but can hardly be very full.'

"The committee trust, moreover, that their hesitation to undertake a very general exhibition will be attributed rather to a natural doubt of their power to accomplish the work with the means at their disposal, than to any disinclination or indifference as to the result. The present call has been issued at the earliest practicable moment, in the hope of securing a sufficiently prompt attention, from all parties, to enable them to do justice to the distant contributors, whom they are still apprehensive of being unable to reach in proper time.

"The general plan of arrangement, and amount of space to be allotted to the several departments and collections, will have to be decided by the end of March; and all the objects to be exhibited must be within reach of the committee by the second or third week of April next.

"In order, therefore, to prevent confusion and disappointment, lists of objects offered for exhibition, and estimates of the amount of space desired for the purpose, will be required as soon as practicable after the publication of this circular.

"Communications addressed to Wm. Pepper, M. D., 1215 Walnut street, and F. F. Maury, M. D., 1218 Walnut street, the Sub-Committee on the Exhibition, will receive immediate attention.

"EDWARD HARTSHORNE, M.D.,
Chairman of the Committee of Arrangements,
1439 Walnut St., Philadelphia.
[Attest, Jan. 20, 1872.]
"D. MURRAY CHESTON, M.D., Secretary, 25 S. 16th St., Philadelphia."

SPRING AND SUMMER MEDICAL INSTRUCTION.—We have received the "*Announcement of the Spring Course for* 1872" in connection with the Rush Medical College. The course will begin on Wednesday, March 6, and will continue until June 26, 1872. The lectures will be given in the Lecture Room of the Cook County Hospital, by the following Instructors:

Prof. J. H. Etheridge, M. D., Instructor in Principles and Practice of Medicine; C. T. Parkes, M. D., Instructor in Anatomy; F. L. Wadsworth, M. D., Instructor in Physiology; E. Fletcher Ingals, M. D., Instructor in Materia Medica; Curtis T. Fenn, M. D., Instructor in Obstetrics; J. E. Owens, M. D., Instructor in Diseases of the Urinary Organs; Prof. E. Powell, M. D., Instructor in Surgery; I. N. Danforth, M. D., Instructor in Pathological Histology; L. W. Case, M. D., Instructor in Chemistry; J. E. O'Brien, M. D., Instructor in Medical Philosophy; W. C. Hunt, M. D., Instructor in Microscopical Anatomy and Use of Microscope.

TERMS: College Matriculation Ticket, $5. This ticket must be obtained for the Spring Course, and will be good one year. Hospital Ticket, $5.

For Circulars, and other information, address the Secretary of the Spring Course, Curtis T. Fenn, M. D., 1155 Michigan avenue.

CHICAGO MEDICAL COLLEGE.—The present regular lecture term, or collegiate year, will close on the second Tuesday in March. The exercises of the public commencement will be held, as usual, on Monday and Tuesday, March 11 and 12. The Valedictory will be delivered by Prof. Thomas Bevan. The usual spring and summer course of instruction in this college will commence on the first day of April, and continue until the first of July following. It will be free to Matriculated students of the college.

DR. A. B. WILLIAMS, of Depere, Wis., sends us the following account of a case of lithotomy, upon which he operated:

The patient, a Hollander 24 years of age, but a short time in this country, had always been troubled, since childhood, in passing water; lately had been able to pass it only as it dribbled away during sleep. Dr. Williams, on being sent for, introduced a sound and struck the stone at once. On the following day he operated by the lateral method, and removed a mulberry calculus one and one-half inches long and one and one-half inches in circumference. The wound healed well, and the patient made a good recovery.

MESSRS. LINDSAY & BLAKISTON announce the publication of a new work on The History of Medicine, by the late Prof. Robley Dunglison, M. D., L.L. D. It comprises the lectures delivered by him, on that subject, at the University of Virginia, collected and arranged from the original manuscript by his son, Richard S. Dunglison, M. D. It will be issued in a small octavo volume of about 250 pages, printed on tinted paper, and handsomely bound in cloth. The price, to subscribers, will be $2.50. Subscriptions received by Lindsay & Blakiston, Philadelphia.

DR. H. N. HURLBUT suggests a solution of acetate of lead, 8 or 10 grains to the pint of water, as an application to relieve the intolerable itching, and also to prevent pitting, in small-pox. He has used it for some four years, and with invariable success.

